# Mental and Physical Health Factors Linking Perceived Control to Brain Health

**DOI:** 10.1002/alz.088226

**Published:** 2025-01-09

**Authors:** Jordan D Palms, Emily P. Morris, Ketlyne Sol, Monica E Nelson, Kiana A. Scambray, Laura B. Zahodne

**Affiliations:** ^1^ University of Michigan, Ann Arbor, MI USA

## Abstract

**Background:**

Perceived control is a psychosocial construct thought to reflect one’s beliefs about their ability to influence life outcomes and includes both internal (self‐efficacy, mastery) and external (structural barriers, constraints) subcomponents. While perceived control has been found to relate to numerous health outcomes such as cardiovascular and mental health, less is known about its impact on brain health. A prior study found that behavioral (i.e., physical activity) and biological (i.e., metabolic systems) health factors mediated the relationship between perceived control and memory (Infurna & Gerstorf, 2013). Furthermore, preliminary analyses indicated greater external control was associated with lower cortical thickness. The current study extends this work by evaluating associations between internal (mastery) versus external (constraints) control and brain health, as well as underlying mechanisms.

**Methods:**

The current study included a subsample of older adults aged 55+ from the Michigan Cognitive Aging Project who completed magnetic resonance imaging (N = 120; Age_mean_ = 62.6; female = 53%; non‐Hispanic Black = 30%). Multiple linear regressions examined associations between Mastery and Constraints subscales from the Perceived Control Scale, and brain health outcomes (cortical thickness composite of nine Alzheimer’s disease signature regions, total gray matter volume, total white matter volume, and white matter hypointensity volume). A mediation analysis explored potential mechanisms of association between perceived control and brain health outcomes through physical (number of chronic illnesses) and mental (depressive symptoms) health factors. Covariates included sociodemographics and total intracranial volume.

**Results:**

Mastery was not associated with brain health outcomes. Constraints were negatively associated with cortical thickness (total effect *b* = ‐.02, 95%CI [‐.03,‐.002]) only. Depressive symptoms fully mediated the relationship between constraints and cortical thickness (indirect effect *b* = ‐.02, 95%CI [‐.03,‐.01]) such that more constraints led to decreased cortical thickness through increased depressive symptoms. No indirect paths through chronic disease burden were found.

**Conclusion:**

These findings suggest that environmental constraints may be an upstream determinant of depressive symptoms, which can lead to negative downstream effects on brain health. Treatment approaches to mental and brain health should address structural barriers in addition to individual factors. Future longitudinal and/or intervention work is needed to confirm the direction of effects, which were based on previous theoretical and empirical work.

